# The Impact of COVID-19 Pandemic on the Diagnosis, Treatment, and Outcomes of Colorectal Cancer in Singapore

**DOI:** 10.3390/medicina61010138

**Published:** 2025-01-16

**Authors:** Hui Lionel Raphael Chen, Piea Peng Lee, Yun Zhao, Wei Hao Caleb Ng, Jiashen Zhao, Yu En Christopher Tan, Bo Jie Sean Loh, Kah-Hoe Pierce Chow, Hiang Khoon Tan, Kwong-Wei Emile Tan

**Affiliations:** 1 Department of Colorectal Surgery, Singapore General Hospital, Singapore 169608, Singapore; 2 Duke-NUS Medical School, Singapore 169857, Singapore; 3 Division of Surgery & Surgical Oncology, National Cancer Centre Singapore, Singapore 168583, Singapore; 4 Group Finance Analytics, Singapore Health Services, Singapore 168753, Singapore; 5 Yong Loo Lin School of Medicine, National University of Singapore, Singapore 117597, Singapore; 6 SingHealth Duke-NUS Global Health Institute, Singapore 168582, Singapore

**Keywords:** COVID-19 pandemic, colorectal cancer, colonoscopy, outcomes, surgery

## Abstract

*Background and Objectives*: During the COVID-19 pandemic, many countries implemented lockdowns and social distancing measures, which may delay the early diagnosis of colorectal cancer (CRC). This study aims to review the impact of the pandemic on the diagnosis and treatment outcomes of CRC. *Materials and Methods*: Patients who underwent colonoscopy or surgery for CRC were included. The study was divided into the pre-COVID-19 (January 2019–January 2020), early COVID-19 (February–May 2020), recovery (June–December 2020), and heightened alert (January–December 2021) periods. Cox regression was used to model the waiting time to colonoscopy. Multivariable logistic regression identified associations between time periods and incidence of CRC diagnosed. The characteristics and outcomes of the surgical procedures that were performed were compared across the time periods. *Results*: A total of 18,662 colonoscopies and 1462 surgical procedures were performed in the study period. Compared to the pre-COVID-19 period, there was a longer time to colonoscopy during the recovery (HR: 0.91; 95% CI: 0.87, 0.94) and heightened alert periods (HR: 0.88; 95% CI 0.85, 0.91). The early COVID-19 (OR: 1.36; 95% CI: 1.04, 1.77) and recovery (OR: 1.20; 95% CI: 1.01, 1.43) periods were associated with higher odds of diagnosing CRC. Compared to the pre-COVID-19 period, there was a higher proportion of ASA 4 patients (4.3% vs. 1.3%; *p* < 0.001) and stage 4 CRC patients (22.2% vs. 16.9%; *p* = 0.001) that required surgery during the heightened alert period. Similarly, there was a higher proportion of emergency surgeries (22% vs. 13.3%; *p* = 0.002); diverting stomas (13.5% vs. 10.5%; *p* = 0.005), and Hartmann’s procedures (4.4% vs. 0.4%; *p* = 0.001) performed during the heightened alert period. *Conclusions*: The pandemic was associated with a higher proportion of metastatic CRC patients requiring surgery. Healthcare policies should facilitate early cancer screening, diagnosis, and treatment to reduce cancer-related morbidity for future pandemics.

## 1. Introduction

Many countries worldwide adopted social distancing measures and nationwide lockdowns during the coronavirus disease 2019 (COVID-19) global pandemic to curb transmission of the virus. The first case of COVID-19 in Singapore was confirmed on 23 January 2020, and local clusters of infection were seen in February 2020. A nationwide lockdown was implemented from April to June 2020 to curb the rise in infections. In December 2020, Singapore commenced the COVID-19 vaccination campaign. However, the emergence of new variants such as delta and omicron in 2021 resulted in intermittent tightening of social distancing measures as the country transitioned to an endemic state [1].

Singapore General Hospital (SGH) is the largest public hospital in Singapore, with 30 clinical disciplines, 43 operating theaters, and 1700 inpatient beds. To reduce the demand on limited healthcare resources, hospitals were advised to reschedule non-urgent elective surgeries and reduce clinic visits. Nonessential elective surgeries were reduced from 7 February 2020, and further reduction was mandated from 7 April 2020 in response to the nationwide lockdown. Emergency and oncologic surgeries were prioritized to ensure timely treatment of patients who were acutely ill or diagnosed with malignancy. While colonoscopies for symptomatic patients continued, colonoscopies for surveillance and colorectal cancer (CRC) screening were postponed. Non-urgent elective surgeries and colonoscopies gradually resumed after the end of the lockdown on 1 June 2020 [2].

The measures implemented may have an impact on cancer care due to the diversion of healthcare resources to treat COVID-19 patients. The pandemic also had an impact on the health-seeking behavior of patients with symptoms of cancer [3]. The delay in CRC screening may result in an increase in the incidence of locally advanced and late-stage disease and emergencies at presentation. In the United Kingdom (UK), national lockdown was introduced in March 2020 with deferment of cancer screening and routine diagnostic work. There was a 50% reduction in elective CRC procedures with a significant increase in mortality from 5.6% to 8.9% after emergency procedures [3]. A population-based modeling study based on the National Health Service (NHS) estimated an additional 1445 to 1563 deaths (15.2–16.6%) up to five years after diagnosis [4]. Most studies examine the impact on the surgical services at the early stages of the pandemic with short-term comparisons before and after national lockdowns [5]. A nationwide study in Japan from Jan 2018 to Dec 2020 observed a temporary decrease in early-stage CRCs after a state of emergency was declared [6]. A multicenter study in Australia reported a higher proportion of emergency surgery performed and metastatic CRCs diagnosed during the COVID-19 period [7]. A study conducted in another hospital in Singapore showed no difference in the stage at presentation and surgical outcomes 6 months after the nationwide lockdown [8]. In Korea, a decreased risk of stage 3 and 4 CRCs was observed during the pandemic period [9]. Given the few studies that assess the longitudinal impact of the pandemic beyond the first year of its onset and the conflicting results, this study aims to review the impact of the COVID-19 pandemic on the diagnosis, treatment, oncologic, and perioperative outcomes of CRC from a tertiary center in Singapore.

## 2. Materials and Methods

### 2.1. Study Design and Participants

This was a retrospective cohort study performed at the Department of Colorectal Surgery, SGH, and included patients who underwent colonoscopy or surgery for colorectal adenocarcinoma from January 2019 to December 2021. The study was conducted in accordance with STROCSS guidelines and was approved by the institutional review board (IRB 2021/2221).

#### 2.1.1. Colonoscopy Service

Patients who underwent colonoscopy after clinical consultation during the study period were included in the study. Patients who underwent surveillance colonoscopies for colonic polyps or CRC were excluded from the study. The outcomes assessed were the time interval to colonoscopy from clinical consultation in our institution, as well as the time interval to surgery from clinical consultation or colonoscopy, the latter of which was measured for patients diagnosed with CRC.

#### 2.1.2. Colorectal Cancer Surgeries 

All patients newly diagnosed with colorectal adenocarcinoma who underwent a surgical procedure were included in the study. Patients who had surgery for benign lesions (e.g., diverticulosis, inflammatory bowel disease) were excluded from the study.

Surgical characteristics considered in our analyses included surgical approach (open vs. laparoscopy vs. robotic), urgency of surgery (elective vs. emergency), surgery intent, and type of surgery. CRC patients may undergo neoadjuvant, adjuvant, or palliative chemotherapy and radiotherapy before surgeries.

#### 2.1.3. Study Outcomes

The primary outcomes of interest were the diagnosis of CRC after colonoscopy and the TNM stage of CRC for patients that underwent surgery. Secondary outcomes included (1) other clinicopathological characteristics of CRC, such as tumor-related complications (e.g., intestinal obstruction or perforation), tumor grade and resection margin status, and (2) perioperative outcomes measured, such as 30-day postoperative mortality and complications and length of hospitalization.

Prioritization of time-sensitive surgeries and postponement of nonurgent elective surgeries occurred from February to May 2020. Nonurgent elective surgeries were allowed from June 2020 onwards. Despite the nationwide vaccination program that began in December 2020, there was intermittent tightening of social distancing measures in 2021 in response to new COVID-19 variants, such as delta and omicron strains [1]. The study period was divided into four segments: the early COVID-19 pandemic period (EC) was between February 2020 and May 2020, during which a national lockdown occurred, while the pre-COVID-19 pandemic period (PC) was between January 2019 and January 2020, the recovery period (RP) was between June 2020 and December 2020, and the heightened alert period (HA) was between January 2021 and December 2021. We analyzed the impact of the pandemic on the diagnosis, treatment, and outcomes of CRC across these four time periods.

### 2.2. Statistical Analysis

The continuous variables were expressed using medians and interquartile ranges, while categorical variables were presented as numbers and percentages. Differences in medians across two or more groups were compared using Mood’s median test or Kruskal–Wallis rank sum test for continuous variables, and proportions were analyzed and compared among groups using the chi-squared test.

Cox regression models were used to model the waiting times between consultation to colonoscopy, colonoscopy to surgery, and consultation to surgery. The events in these models were defined as either the first colonoscopy or surgery performed for the patient. Time to event was faster if hazard ratio was more than one.

Multivariable logistic regression analyses were performed to evaluate associations between the time periods and the incidence of CRC diagnosed, stage four CRC, or post-operative complications.

All statistical tests were two-sided. A *p*-value of less than 0.05 was considered statistically significant for all tests. A missing value for any variable is omitted in our analyses. All statistical analyses were performed using R version 4.3.1 within RStudio software version 2023.06.0 Build 421 (R Foundation for Statistical Computing, Vienna, Austria).

## 3. Results

### 3.1. Characteristics of Colonoscopy Service over Time

**Figure 1:**
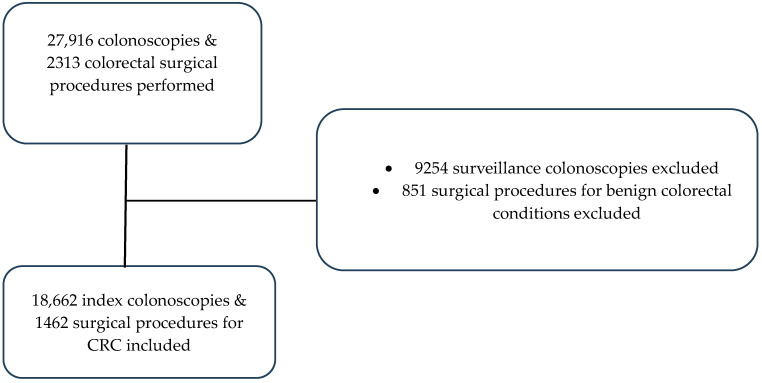
Flowchart of patients identified and excluded for inclusion in the study.

A total of 18,662 colonoscopies were performed in the study period (Figure 1). The median number of colonoscopies performed per month were 490, 270, 514, and 630 during the pre-COVID-19, early COVID-19, recovery, and heightened alert periods, respectively (Table 1).

Compared to the pre-COVID-19 period, the proportion of CRCs diagnosed after colonoscopy was higher in the early COVID-19 period (9.3% vs. 7.0%; *p* < 0.001) but lower during the heightened alert period (5.6% vs. 7.0%; *p* < 0.001).

Compared to the pre-COVID-19 period, there was no difference in time from consultation to colonoscopy during the early COVID-19 period. However, there was a longer time from consultation to colonoscopy during the recovery (HR: 0.91; 95% CI: 0.87, 0.94) and heightened alert periods (HR: 0.88; 95% CI: 0.85, 0.91)(Table 2).

There was no difference in the waiting time from colonoscopy to CRC surgery in the early COVID-19 and recovery periods, but CRC surgery was performed earlier during the heightened alert period (HR: 1.45; 95% CI: 1.22, 1.72) (Table 2).

After adjusting for age, type of priority for colonoscopy, type of consultation, and time periods with multivariable logistic regression, colonoscopy performed in early COVID-19 (OR = 1.36; 95% CI 1.04, 1.77) and recovery periods (OR = 1.2; 95% CI 1.01, 1.43) were more likely to diagnose CRC (Table 3).

### 3.2. Characteristics of the Surgical Care Service over Time

A total of 1462 surgical procedures were performed for newly diagnosed CRC in the study period (Figure 1), and the median number of surgical procedures performed per month were 38, 38, 44, and 46 during the pre-COVID-19, early COVID-19, recovery, and heightened alert periods, respectively. Table 4 presents the demographics, clinico-pathological characteristics and perioperative outcomes of patients.

Compared with the pre-COVID-19 period, there was a significantly higher proportion of ASA 4 patients (4.3% vs. 1.3%; *p* < 0.001), emergency surgeries performed (22% vs. 13.3%; *p* = 0.002), diverting stomas created (13.5% vs. 10.3%; *p* = 0.005), and Hartmann procedures performed (4.4% vs. 0.4%; *p* = 0.001) in the heightened alert period. Similarly, there were significantly higher proportions of stage four CRCs diagnosed (22.2% vs. 16.9%; *p* = 0.001), lymphovascular invasion (35.3% vs. 26.7%; *p* = 0.02) identified on pathology, and a higher proportion of palliative chemotherapy administered (12.6% vs. 9.3%; *p* = 0.02) during the heightened alert period when compared with pre-COVID-19 period.

After adjusting for presence of stoma and tumor-associated complications with multivariable logistic regression, surgery performed during the heightened alert period was associated with stage four CRC (OR = 1.54; 95% CI 1.06, 2.24) (Table 5).

Across the four time periods, there was no significant difference in the proportion of R0 resections, 30-day post-operative hospitalization length of stay, and readmission within 30 days (Table 4 and Table 6). Compared to the pre-COVID-19 period, there were lower postoperative 30-day mortality rates from the early COVID-19 (0.7% vs. 3%) to the heightened alert period (1.1% vs. 3%). However, there was a significantly higher proportion of postoperative complications in the early COVID-19 period (34.3% vs. 25.5%; *p* = 0.04) when compared to the pre-COVID-19 period. With regard to the type of postoperativecomplications, there was a significantly higher proportion of cerebrovascular events (1.3% vs. 0%; *p* = 0.05) in the heightened alert period compared to the pre-COVID-19 period (Table 6).

After adjusting for age, ASA, emergency surgery, surgical approach, stoma, tumor complications, and TNM stage with multivariable logistic regression, there was no difference in—postoperative complications during the early COVID-19 and recovery periods compared to the pre-COVID-19 period (Table 7).

## 4. Discussion

This is the first study in Singapore that investigated the impact of the COVID-19 pandemic two years after its onset on the diagnosis and surgical outcomes of CRC patients at a high-volume tertiary referral center in Singapore. Despite prioritizing cancer diagnosis and treatment at the onset of the pandemic, during the heightened alert period, there was a higher proportion of stage 4 CRC patients that required palliative surgery.

Most studies showed that the diagnosis of CRC has significantly reduced during the pandemic due to the reduction in CRC screening programs and redistribution of healthcare resources [5]. In our study, there was no difference in time to colonoscopy in early COVID-19 and recovery periods. However, the time to colonoscopy was slower in the heightened alert period. Interestingly, patients who had colonoscopies performed during the early COVID-19 and recovery periods were more likely to be diagnosed with CRC. This observation reflects the healthcare policy that allowed surgeons to be judicious in postponing colonoscopies that were not urgent and prioritizing patients who were clinically assessed as requiring early colonoscopy. Among patients diagnosed with CRC from colonoscopy, surgery was performed faster in the heightened alert period. Robotic surgery continued during the early COVID-19 period when compared to the pre-COVID-19 period (15.3% vs. 11.4%). As nonurgent elective surgeries were rescheduled, this allowed for the available operating theaters to be utilized for cancer surgeries and contributed to earlier operations for CRC patients diagnosed during the heightened alert period. This also allowed for sufficient hospital capacity to accommodate minimally invasive and robotic rectal cancer surgery. Previously, there were concerns that laparoscopy and robotic surgery may aerosolize and transmit COVID-19 viral particles. However, robotic surgery may be potentially advantageous in minimizing viral transmission with increased surgeon-patient interface via console operating and shorter length of hospitalization stay [10,11]. Recent studies conclude that it is safe and feasible for robotic surgery to continue during the pandemic if there are available resources [12,13].

The monthly median numbers of colonoscopies and surgeries performed were higher in the recovery and heightened alert periods at our institution. In Singapore, regional hospitals at full capacity with the influx of COVID-19 patients would divert patients to tertiary hospitals with additional capacity for the treatment of non-COVID-19 patients. Patients unable to obtain early specialist consultations at peripheral hospitals were likely to seek treatment at tertiary hospitals. SGH, the largest public tertiary hospital in the central region of Singapore, receives most referrals from regional hospitals for subspecialty and multidisciplinary care. Given its larger capacity and infrastructure, the hospital can accommodate additional non-COVID-19 patients during the pandemic diverted from regional hospitals. While this may have contributed to higher operative, endoscopic, and emergency workloads seen in the recovery and heightened alert periods, hospital diversions may also contribute to delays in the presentation and diagnosis of CRC.

Despite the higher number of colonoscopies and surgeries performed in the heightened alert period, the number of CRCs diagnosed by colonoscopy was lower in comparison to the number of operative procedures performed for CRC. In our institution, colonoscopies can be performed by a gastroenterologist, colorectal, or general surgeon. This study only presented the number of colonoscopies performed by colorectal surgeons in the hospital. It is possible that there were CRCs diagnosed by gastroenterologists and general surgeons on colonoscopy that eventually needed an operative procedure. CRC patients with local complications associated with the primary tumor, like sealed perforation or obstruction, may not undergo preoperative colonoscopy prior to surgery, and these complications may also contribute to the difference in the number of CRCs diagnosed via colonoscopy and operative procedures performed.

Most studies demonstrated an increase in urgent referrals of obstruction, perforation, or advanced stages of cancer after the onset of the pandemic [5,14]. However, a study from another hospital in Singapore showed that CRC TNM stage, severity at presentation, and operative outcomes were similar 6 months before and after nationwide lockdown [8]. This was also observed in a multicenter cohort study in France, which showed that tumor stage at presentation and emergency surgeries for CRC did not differ between 2020 and 2018 to 2019 [15]. Studies from the Netherlands Cancer Registry and Korea also demonstrated limited impact on CRC care [9,16,17]. The limited impact of the pandemic on CRC diagnosis and treatment in Korea was likely due to the absence of a national or regional lockdown policy [9]. In the Netherlands, the reduced median time between CRC diagnosis and initial treatment was due to the continued prioritization of CRC surgical procedures throughout the pandemic [17].

In this study, there was a higher proportion of stage four CRC patients in the heightened alert period who underwent an operative procedure. Patients were more critically ill with ASA 4 at presentation requiring emergency surgery. This may account for the higher proportion of patients who needed Hartmann’s procedure or a diverting stoma in the heightened alert period. The higher proportion of patients who needed palliative chemotherapy was due to the increased proportion of stage four CRCs. Despite a higher proportion of emergency surgeries, ASA 4 and stage four patients, the postoperative complications and length of stay were comparable across all the time periods. The lower postoperative mortality seen in the heightened alert period compared to the pre-COVID-19 period can be attributed to a lower proportion of rectal cancer surgical resections performed. Rectal resections for cancer have a mortality rate of 2.6 to 5.3% [18,19]. Less curative rectal resections performed in the heightened alert period may contribute to the lower mortality rate.

Most studies attributed stage migration of CRC to delays in CRC screening and colonoscopies. Our observations concur with other multicenter studies in Italy, Japan, and Australia that demonstrated a significant association between the COVID-19 pandemic and more advanced stage and metastatic CRC at diagnosis [6,7,20,21]. Studies in the UK report diagnostic delays of 5.4% to 26% [22]. 79.3% of surgeons in the UK delayed their surgeries, and 10.3% stopped their surgeries [23]. There was reduced short-term survival for colorectal cancer observed in population-based cohort studies in Belgium and the UK after the pandemic’s onset [24,25].

Individuals may weigh the risk of COVID-19 transmission more heavily than potential benefits of early cancer detection or prevention and will postpone cancer screening until the risk of cancer outweighs the risk of COVID-19 infection [26,27]. Population-based and community studies suggest that people were less likely to seek medical help for their symptoms, were fearful of catching COVID-19, and were less likely to be referred for CRC [28,29,30].

As only CRC patients who underwent surgery were included in this study, CRC patients who did not require surgery were usually metastatic at presentation. Systemic chemotherapy is the main treatment for metastatic CRC patients with minimal symptoms from the primary tumor and acceptable performance status. However, 9% to 29% of metastatic CRC patients on chemotherapy will require palliative surgical intervention [31,32]. The proportion of stage four CRCs that needed an operative procedure in our institution in the heightened alert period was 22%, and this approximated the Singapore Cancer Registry data of 25% to 27% of stage four CRCs nationally [33,34,35]. While there may not be a significant change in TNM stage distribution nationally, stage four CRC patients were more likely to require surgical intervention like palliative stoma or resection prior to palliative chemotherapy in 2021 than in 2020 and 2019. It is possible that due to the pandemic, patients with undiagnosed stage four CRC may have avoided healthcare institutions and only presented later to hospitals with more symptoms, heavier disease burden, and increased luminal stenosis on colonoscopy, therefore requiring a palliative operative procedure.

### 4.1. Strengths & Limitations

While this is a single-center retrospective study limiting the generalization of the findings, the study was based on the high-volume and largest tertiary public hospital in Singapore, which may represent the country’s national CRC trends.

Only the clinical data of CRC patients that underwent surgery were collected. Malignant colorectal polyps removed endoscopically and on surveillance, as well as metastatic CRC patients on palliative chemotherapy, were not reflected in this study. According to the Singapore Cancer Registry 2020 report, from 2018 to 2020, the proportions of stage four CRCs among males and females were 25.4% and 27.1%, respectively [34]. In the subsequent Singapore Cancer Registry 2021 report from 2018 to 2021, the proportions of stage four CRCs among males and females were 25.8% and 26.9%, respectively [35]. These findings suggest that there were no significant shifts towards a higher proportion of stage four CRCs in 2021 nationally [33,34,35]. However, our study suggests that during the heightened alert period, more stage four CRC patients required palliative surgical interventions prior to chemotherapy.

The strength of our study compared to earlier studies was that we examined the surgical outcomes of CRC patients diagnosed one to two years after the onset of the pandemic [8]. This allows for evaluation of the longitudinal impact of the changing national lockdown policies during the first two years of the pandemic on CRC care at our institution [1].

### 4.2. Implications for Healthcare Policies During Future Pandemics

It is imperative that hospital policies should continue to facilitate CRC screening and expedite investigation of patients with symptoms associated with CRC during future pandemics. Organized cancer screening programs should continue to promote the use of home-based screening tests such as the fecal immunohistochemical test (FIT) to prioritize patients for colonoscopy without requiring any clinic visits [36]. Public health campaigns can continue to encourage individuals to seek early treatment if they develop symptoms of CRC even during a pandemic. Efforts should be made by hospitals to ensure continued and early access to colonoscopies for symptomatic and FIT-positive patients. The adoption of telemedicine and digital technology during future pandemics may facilitate CRC screening and ensure access to early colonoscopy through virtual consultations with their healthcare providers. Patients with risk factors or symptoms suggestive of CRC can be prioritized through telehealth consultations for earlier colonoscopies [37,38]. Finally, colonoscopies can be performed during a pandemic if endoscopists wear personal protective equipment (PPE), which does not affect the quality indicators for endoscopy. Mandatory use of PPE and stringent sanitation procedures will ensure timely diagnosis of CRC with colonoscopy while minimizing viral transmissions [39,40].

## 5. Conclusions

Despite a healthcare policy that ensured that cancer diagnosis and surgeries were prioritized amidst the COVID-19 pandemic, there was a higher proportion of stage four CRC that needed palliative surgical intervention. Our study highlights the importance of increased public health awareness campaigns to encourage patients to seek timely treatment for non-COVID-19-related symptoms during a pandemic. Healthcare policy should continue to facilitate early diagnosis and treatment of CRC and reduce cancer-related morbidity in the face of future pandemics..

## Figures and Tables

**Table 1:** Characteristics of patients that underwent colonoscopy after colorectal specialist consultation from 2019 to 2021 (*n* = 18,662).

Variable	Pre-COVID-19 (PC)	Early COVID-19 (EC)	Recovery Period (RP)	Heightened Alert Period (HA)	*p*-Value
	(*n* = 6496)	(*n* = 1036)	(*n* = 3686)	(*n* = 7444)	
Number of colonoscopes per month (median)	490	270	514	630	0.26
Age, median (years, IQR)	61 (IQR 17)	61 (IQR 16)	61 (IQR 18)	61 (IQR 18)	0.35
Gender (*n*, %)					
Male	3222 (49.6)	477 (46.0)	1778 (48.2)	3602 (48.4)	0.13
Female	3274 (50.4)	559 (54.0)	1908 (51.8)	3842 (51.6)	0.13
Urgency of colonoscopy (*n*, %)					
Elective	6327 (97.4)	1007 (97.2)	3628 (98.4)	7308 (98.2)	<0.001
Emergency	169 (2.6)	29 (2.8)	58 (1.6)	136 (1.8)	<0.001
Type of colorectal specialist consultation (*n*, %)					
Outpatient	6088 (93.7)	968 (93.4)	3452 (93.7)	7084 (95.2)	<0.001
Inpatient	408 (6.3)	68 (6.6)	234 (6.3)	360 (4.8)	<0.001
Post-Colonoscopy Diagnosis (*n*, %)					
Benign	6042 (93.0)	940 (90.7)	3397 (92.2)	7026 (94.4)	<0.001
Malignant	454 (7.0)	96 (9.3)	289 (7.8)	418 (5.6)	<0.001

**Table 2:** Cox regression analysis of waiting time to colonoscopy and colorectal cancer surgery.

	Time from Consultation to Colonoscopy						Time from Colonoscopy to Surgery					
Variable	Univariate			Multivariable			Univariate			Multivariable		
	HR	95% CI	*p*-Value	HR	95% CI	*p*-Value	HR	95% CI	*p*-Value	HR	95% CI	*p*-Value
Urgency of colonoscopy												
Emergency vs. Elective	1.92	(1.73, 2.12)	<0.001	1.54	(1.39, 1.71)	<0.001	1.26	(1.01, 1.58)	0.04	0.971	(0.765, 1.23)	0.81
Type of colorectal specialist consultation												
Inpatient vs. Outpatient	2.3	(2.16, 2.45)	<0.001	2.17	(2.03, 2.31)	<0.001	1.6	(1.38, 1.85)	<0.001	1.63	(1.4, 1.91)	<0.001
Post-Colonoscopy Diagnosis												
Malignant vs. Benign	1.35	(1.27, 1.43)	<0.001	1.3	(1.23, 1.37)	<0.001						
Time period type												
Early COVID-19 vs. Pre-COVID-19	0.9995	(0.936, 1.07)	0.99	0.978	(0.916, 1.04)	0.51	1.14	(0.877, 1.47)	0.34	1.12	(0.862, 1.45)	0.40
Recovery vs. Pre-COVID-19	0.906	(0.87, 0.944)	<0.001	0.906	(0.87, 0.944)	<0.001	1.17	(0.967, 1.41)	0.11	1.13	(0.938, 1.37)	0.20
Heightened Alert vs. Pre-COVID-19	0.87	(0.841, 0.899)	<0.001	0.875	(0.846, 0.905)	<0.001	1.42	(1.19, 1.69)	<0.001	1.45	(1.22, 1.72)	<0.001

**Table 3:** Multivariable logistic regression analyses of factors associated with colorectal cancer diagnoses after colonoscopy.

Variable	Univariate			Multivariable		
	OR	95% CI	*p*-Value	OR	95% CI	*p*-Value
Age	1.03	(1.02, 1.04)	<0.001	1.02	(1.02, 1.03)	<0.001
Type of priority for colonoscopy						
Emergency vs. Elective	3.8	(2.86, 5)	<0.001	0.853	(0.617, 1.18)	0.34
Type of colorectal specialist consultation						
Inpatient vs. Outpatient	6.39	(5.42, 7.54)	<0.001	5.75	(4.77, 6.94)	<0.001
Time period type						
Early COVID-19 vs. Pre-COVID-19	1.38	(1.06, 1.78)	0.02	1.36	(1.04, 1.77)	0.02
Recovery vs. Pre-COVID-19	1.19	(1.004, 1.41)	0.05	1.20	(1.01, 1.43)	0.04
Heightened alert vs. Pre-COVID-19	0.823	(0.706, 0.958)	0.01	0.871	(0.745, 1.02)	0.08

**Table 4:** Clinicopathological characteristics of colorectal cancer patients that underwent surgery from 2019 to 2021 (*n* = 1462).

Characteristics	Pre-COVID-19	Early COVID-19	Recovery Period	Heightened Alert Period	*p*-Value
	(*n* = 474)	(*n* = 137)	(*n* = 311)	(*n* = 540)	
Number of surgeries performed per month (median)	38	38	44	46	0.39
Gender					
Female	205 (43.2)	65 (47.4)	145 (46.6)	241 (44.6)	0.74
Male	269 (56.8)	72 (52.6)	166 (53.4)	299 (55.4)	>0.74
ASA Score					
1	17 (3.6)	5 (3.6)	8 (2.6)	6 (1.1)	0.059
2	300 (63.6)	87 (63.5)	189 (61.0)	316 (58.5)	0.38
3	149 (31.6)	44 (32.1)	112 (36.1)	195 (36.1)	0.38
4	6 (1.3)	1 (0.7)	1 (0.3)	23 (4.3)	<0.001
Urgency of surgery					
Elective	411 (86.7)	119 (86.9)	255 (82.0)	421 (78.0)	0.002
Emergency	63 (13.3)	18 (13.1)	56 (18.0)	119 (22.0)	0.002
Surgical approach					
Open	140 (29.5)	35 (25.5)	81 (26.0)	163 (30.2)	0.48
Laparoscopic	280 (59.1)	81 (59.1)	205 (65.9)	339 (62.8)	0.23
Robotic	54 (11.4)	21 (15.3)	25 (8.0)	38 (7.0)	0.007
Type of surgery performed					
Abdominoperineal Resection	14 (3.0)	9 (6.6)	15 (4.8)	13 (2.4)	0.05
Diverting Colostomy/Ileostomy	49 (10.3)	6 (4.4)	25 (8.1)	73 (13.5)	0.005
Extended/High Anterior Resection	153 (32.3)	42 (30.7)	95 (30.6)	166 (30.7)	0.95
Extended/Right Hemicolectomy	93 (19.6)	30 (21.9)	76 (24.5)	121 (22.4)	0.43
Hartmann Procedure	2 (0.4)	4 (2.9)	10 (3.2)	24 (4.4)	0.001
Left Hemicolectomy	29 (6.1)	5 (3.6)	14 (4.5)	23 (4.3)	0.47
Low Anterior Resection	59 (12.4)	18 (13.1)	34 (11.0)	59 (10.9)	0.8
Panproctocolectomy	1 (0.2)	1 (0.7)	2 (0.6)	2 (0.4)	0.74
Subtotal Colectomy	5 (1.1)	1 (0.7)	2 (0.6)	5 (0.9)	0.94
Total Colectomy	7 (1.5)	1 (0.7)	2 (0.6)	10 (1.9)	0.46
Transanal	5 (1.1)	0 (0.0)	1 (0.3)	4 (0.7)	0.47
Ultra-Low Anterior Resection	56 (11.8)	20 (14.6)	34 (11.0)	38 (7.0)	0.015
Location of tumor					
Colon	288 (60.8)	80 (58.4)	186 (59.8)	347 (64.3)	0.43
Rectum	186 (39.2)	57 (41.6)	125 (40.2)	193 (35.7)	0.43
Local complications associated with tumor					
Abscess/Sealed Perforation	12 (2.5)	5 (3.6)	11 (3.5)	12 (2.2)	0.62
Fistula	4 (0.8)	1 (0.7)	3 (1.0)	2 (0.4)	0.72
Gross Perforation	10 (2.1)	3 (2.2)	4 (1.3)	9 (1.7)	0.83
Intussuscepted	1 (0.2)	0 (0.0)	1 (0.3)	6 (1.1)	0.16
Obstructed	69 (14.6)	16 (11.7)	50 (16.1)	83 (15.4)	0.66
pT stage					
T1	59 (13.6)	23 (17.7)	32 (11.1)	52 (11.1)	0.18
T2	63 (14.5)	15 (11.5)	42 (14.5)	66 (14.1)	0.85
T3	208 (47.8)	59 (45.4)	153 (52.9)	234 (50.1)	0.42
T4	105 (24.1)	33 (25.4)	62 (21.5)	115 (24.6)	0.74
pN stage					
N0	236 (54.5)	81 (61.8)	157 (54.3)	236 (50.8)	0.15
N1	197 (45.5)	50 (38.2)	132 (45.7)	229 (49.2)	0.15
TNM stage					
Stage 1	98 (20.7)	34 (24.8)	60 (19.3)	87 (16.1)	0.08
Stage 2	111 (23.4)	39 (28.5)	89 (28.6)	136 (25.2)	0.35
Stage 3	185 (39.0)	47 (34.3)	123 (39.5)	197 (36.5)	0.61
Stage 4	80 (16.9)	17 (12.4)	39 (12.5)	120 (22.2)	0.001
Resection Margin					
R0	466 (98.5)	132 (96.4)	302 (97.1)	405 (96.9)	0.32
R1	7 (1.5)	5 (3.6)	9 (2.9)	13 (3.1)	0.32
Perineural invasion present	117 (27.5)	19 (15.0)	68 (24.7)	107 (23.1)	0.03
Lymphovascular invasion present	113 (26.7)	33 (26.0)	77 (27.9)	164 (35.3)	0.02
Chemotherapy					
Neoadjuvant	56 (11.8)	14 (10.2)	35 (11.4)	47 (8.7)	0.39
Adjuvant	174 (36.7)	39 (28.5)	110 (35.7)	188 (34.9)	0.36
Palliative	44 (9.3)	8 (5.8)	22 (7.1)	68 (12.6)	0.02
Radiotherapy					
Neoadjuvant	40 (8.4)	14 (10.2)	31 (10.1)	37 (6.9)	0.34
Adjuvant	8 (1.7)	3 (2.2)	5 (1.6)	12 (2.2)	0.90
Palliative	9 (1.9)	0 (0.0)	5 (1.6)	11 (2.0)	0.42

**Table 5:** Multivariable logistic regression of factors associated with stage four colorectal cancer.

Independent Variables	Univariate			Multivariable		
	OR	95% CI	*p*-Value	OR	95% CI	*p*-Value
Presence of stoma						
Stoma vs. No stoma	4.24	(3.08, 5.82)	<0.001	3.58	(2.58, 4.97)	<0.001
Tumor complications						
Complication vs. None	3.45	(2.49, 4.78)	<0.001	2.82	(2.00, 3.99)	<0.001
Time period type						
Early COVID-19 vs. Pre-COVID-19	0.754	(0.397, 1.43)	0.39	0.696	(0.355, 1.37)	0.29
Recovery vs. Pre-COVID-19	0.805	(0.512, 1.27)	0.35	0.792	(0.490, 1.28)	0.34
Heightened alert vs. Pre-COVID-19	1.56	(1.09, 2.22)	0.02	1.54	(1.06, 2.24)	0.03

**Table 6:** Perioperative outcomes of colorectal cancer patients that underwent surgery from 2019 to 2021.

Characteristics	Pre-COVID-19	Early COVID-19	Recovery Period	Heightened Alert Period	*p*-Value
	(*n* = 474, median = 38)	(*n* = 137, median = 38)	(*n* = 311, median = 44)	(*n* = 540, median = 46)	
Post-operative 30-day mortality (*n* dead, %)	14 (3.0)	1 (0.7)	2 (0.7)	6 (1.1)	0.03
Post-operative length of stay, median (days, IQR)	6 (IQR 4)	6 (IQR 5)	6 (IQR 6)	6 (IQR 5)	0.13
Post-operative complications	121 (25.5)	47 (34.3)	80 (25.7)	121 (22.4)	0.04
30-day readmissions	41 (8.6)	17 (12.4)	34 (10.9)	69 (12.8)	0.20
Type of post-operative complications					
Surgical site infection	52 (11.0)	21 (15.3)	26 (8.4)	41 (7.6)	0.03
Pneumonia	14 (3.0)	6 (4.4)	11 (3.5)	14 (2.6)	0.69
Pulmonary embolism	3 (0.6)	2 (1.5)	2 (0.6)	2 (0.4)	0.55
Deep vein thrombosis	4 (0.8)	1 (0.7)	2 (0.6)	1 (0.2)	0.53
Renal insufficiency	6 (1.3)	1 (0.7)	3 (1.0)	10 (1.9)	0.62
Myocardial infarction	3 (0.6)	1 (0.7)	4 (1.3)	3 (0.6)	0.67
Blood transfusions within 72 h after surgery	58 (12.2)	20 (14.6)	34 (10.9)	49 (9.1)	0.20
Anastomotic leak	11 (2.3)	4 (2.9)	1 (0.3)	8 (1.5)	0.10
Cerebrovascular accident	0 (0.0)	2 (1.5)	1 (0.3)	7 (1.3)	0.045

**Table 7:** Multivariable logistic regression of association of factors associated with postoperative complications after surgery.

Independent Variables	Univariate			Multivariable		
	OR	95% CI	*p*-Value	OR	95% CI	*p*-Value
Age (years)	1.02	(1.01, 1.04)	0.001	1.01	(0.995, 1.03)	0.18
ASA Score						
2 vs. 1	1.62	(0.56, 4.7)	0.37	1.28	(0.371, 4.43)	0.70
3 vs. 1	3.73	(1.28, 10.85)	0.02	2.6	(0.731, 9.25)	0.14
4 vs. 1	11.2	(3.02, 41.6)	<0.001	2.85	(0.564, 14.38)	0.21
Urgency of surgery						
Elective (reference)						
Emergency	2.62	(1.92, 3.59)	<0.001	1.01	(0.577, 1.76)	0.98
Surgery approach						
Open vs. Lap	3.35	(2.51, 4.49)	<0.001	2.47	(1.66, 3.68)	<0.001
Robotic vs. Lap	1.63	(1.03, 2.59)	0.04	1.21	(0.712, 2.07)	0.48
Surgery stoma						
Stoma vs. No Stoma	2.37	(1.81, 3.11)	<0.001	2.05	(1.44, 2.91)	<0.001
Local complications associated with tumor						
Complications vs. None	3.31	(2.45, 4.47)	<0.001	2.52	(1.55, 4.09)	<0.001
Final TNM stage						
Stage 2 vs. Stage 1	2.39	(1.5, 3.82)	<0.001	1.63	(0.953, 2.79)	0.07
Stage 3 vs. Stage 1	2.46	(1.58, 3.84)	<0.001	1.78	(1.08, 2.95)	0.02
Stage 4 vs. Stage 1	3.18	(1.95, 5.17)	<0.001	2.14	(1.12, 4.07)	0.02
Time period type						
Early COVID-19 vs. Pre-COVID-19	1.58	(0.994, 2.52)	0.05	1.48	(0.841, 2.59)	0.18
Recovery vs. Pre-COVID-19	1.01	(0.703, 1.46)	0.95	0.78	(0.496, 1.23)	0.28
Heightened alert vs. Pre-COVID-19	0.87	(0.629, 1.2)	0.40	0.523	(0.342, 0.799)	0.003

## Data Availability

Not available for public access.
